# AKT1 genotype moderates the acute psychotomimetic effects of naturalistically smoked cannabis in young cannabis smokers

**DOI:** 10.1038/tp.2015.219

**Published:** 2016-02-16

**Authors:** C J A Morgan, T P Freeman, J Powell, H V Curran

**Affiliations:** 1 Clinical Psychopharmacology Unit, University College London, London, UK; 2 Psychopharmacology and Addiction Research Centre, University of Exeter, Exeter, UK; 3 Basic and Clinical Neurosciences, Institute of Psychiatry, Kings College London, London, UK

## Abstract

Smoking cannabis daily doubles an individual's risk of developing a psychotic disorder, yet indicators of specific vulnerability have proved largely elusive. Genetic variation is one potential risk modifier. Single-nucleotide polymorphisms in the AKT1 and catechol-O-methyltransferase (COMT) genes have been implicated in the interaction between cannabis, psychosis and cognition, but no studies have examined their impact on an individual's acute response to smoked cannabis. A total 442 healthy young cannabis users were tested while intoxicated with their own cannabis—which was analysed for delta-9-tetrahydrocannbinol (THC) and cannabidiol content—and also ±7 days apart when drug-free. Psychotomimetic symptoms and working memory were assessed on both the sessions. Variation at the rs2494732 locus of the AKT1 gene predicted acute psychotic response to cannabis along with dependence on the drug and baseline schizotypal symptoms. Working memory following cannabis acutely was worse in females, with some suggestion of an impact of COMT polymorphism on working memory when drug-free. These findings are the first to demonstrate that AKT1 mediates the acute response to cannabis in otherwise healthy individuals and implicate the AKT1 pathway as a possible target for prevention and treatment of cannabis psychosis.

## Introduction

The finding that cannabis use is associated with an elevated risk of schizophrenia^1^ has captured considerable attention from mental health professionals and the media alike. However, only a very small minority of individuals who use the drug will develop psychotic symptoms. Nevertheless, as the use of cannabis represents possibly the most modifiable environmental risk factor for schizophrenia, much effort has been devoted to ways of identifying those who are vulnerable to these effects of the drug. One avenue of investigation has been genetic susceptibility. Genetic risk of a psychotic disorder may be suggested by the increased sensitivity to the psychotomimetic effects of cannabis in individuals with a family history of schizophrenia:^2^ Positive schizotypal symptoms from recent cannabis use were 15 times greater in unaffected siblings of patients with schizophrenia compared with controls. However, the genes that underpin this susceptibility remain unclear. An initial study suggested that a functional polymorphism in the catechol-O-methyltransferase (COMT) gene (Val158Met) may mediate this sensitivity,^3^ but subsequent larger-scale studies failed to replicate this finding.^4^

Recently a new candidate for a gene × cannabis interaction has been identified in the AKT1 gene. AKT1 is a promising target as delta-9-tetrahydrocannbinol (THC) has been shown to activate AKT1 *in vitro*^5^ and *in vivo*; THC administration to mice activates AKT1 in the hippocampus, striatum and cerebellum.^6^ The AKT1 gene codes for a protein kinase that forms part of the striatal dopamine receptor signalling cascade^7^ and hence has a plausible biological mechanism for interacting with cannabis to confer an increased risk of schizophrenia. Several studies have begun to support this link. In 801 patients with schizophrenia and 740 unaffected siblings, of 152 candidate single-nucleotide polymorphisms, the AKT1 rs2494732 polymorphism was the only one to emerge as interacting with cannabis.^8^ Carriers of two copies of the C allele of the rs2494732 (single-nucleotide polymorphism) of the AKT1 gene were at a twofold greater risk of being diagnosed with a psychotic disorder (patients) or having greater schizotypy (siblings) if they had used cannabis. This gene × environment interaction was replicated in a subsequent study of 489 patients with first-episode psychosis where carriers of the C/C genotype with a history of cannabis use were again shown to have greater than twofold risk of developing a psychotic disorder.^9^ Cognitive impacts of this polymorphism have also been observed with cannabis-using psychotic patients who are carriers of the AKT1 rs2494732 C/C genotype performing more poorly on a task of sustained attention than T/T carriers.^10^

Although previous studies have suggested that the acute effects of cannabis are mediated by the AKT1 polymorphism, they have not assessed acute effects at the time of smoking cannabis and relied instead on retrospective reports. Further, previous studies were all of patients either with psychotic disorders or at a familial risk. It is important to know whether this polymorphism is also indicative of risk in individuals with no familial history of schizophrenia.

The current study, therefore, set out to examine the gene × cannabis use interaction in a group of healthy young cannabis users, with no family history of schizophrenia, assessed both at the time of smoking cannabis and when non-intoxicated. We assessed the impact of the two loci that have been previously demonstrated to have an interaction between psychotic symptoms and cannabis: the functional Valine158Methionine (Val158Met) polymorphism of the COMT gene and AKT1 rs2494732. Given these genotypes have been shown to interact with working memory,^11, 12^ we included a measure of this in our study. Our novel naturalistic design allowed us to assess (1) acute psychotic symptoms, (2) cognitive function and (3) level of THC, the main active ingredient in the cannabis smoked at the time of ingestion.

## Materials and methods

### Participants

A total sample of 422 cannabis users (308 males, 114 females), aged between 16 and 23 years was used for data analysis. Inclusion criteria required that all the participants abstain from drinking alcohol and smoking cannabis 24 h before each testing day. Sample size was determined from previous studies of COMT.^4^ Cannabis users were required to currently use cannabis at least once per month, have English as a native language, no learning disabilities or major physical or psychiatric health problems, have normal or corrected-normal vision and no history of psychosis either personally or in a first-degree relative. All the participants gave written, witnessed, informed consent on both the test days. The study was approved by the institutional (UCL) ethics committee, and its aims were supported by the UK Home Office. Given the ethical concerns of studying active cannabis use, the volunteer information sheet stated that the researchers did not condone the use of cannabis, and following testing, participants were provided with a cannabis information leaflet, which included a helpline number to contact should they want advice on their drug use.

### Procedure

All the participants were tested on two separate test days, in their own homes. The order of testing (non-intoxicated and intoxicated) was counterbalanced, with the second test day being a week after the first (±1 day). Test versions of the *N*-back task were also balanced across the two test days. Participants were required to abstain from alcohol and cannabis 24 h before each test day.

On the non-intoxicated day following consent, demographic data were collected (self-reported ethnicity, age, gender, employment status, level of educational attainment, drug use history). Hair analysis was used to verify drug use history. A urine drug screen was performed to confirm abstinence from other drugs (ketamine, opiates, cocaine, amphetamine, methamphetamines/ecstasy and benzodiazepines). After that, participants completed the Cannabis Experience Questionnaire (modified version:^13^ Schizotypyal Personality Questionnaire (SPQ), Psychotomimetic States Inventory and *N*-back and other measures reported elsewhere.^14, 15^

On the intoxicated day, all the participants were individually tested in the same location. Following consent, a urine sample was taken for urinalysis before the smoking of cannabis. All the participants then prepared their cannabis for consumption; at this point, the participants also provided a 0.3 g sample of their own cannabis for cannabinoid analysis by the Forensic Science Service, UK. The participant then smoked cannabis in front of the experimenter who told participants to smoke at their usual inhalation rate, and to smoke as much as they would normally do to feel ‘stoned.' At this point, the testing began. Following testing on the second day, the participants were fully debriefed and reimbursed for their time.

### Measures

Psychotomimetic States Inventory^16^—A 48-item questionnaire, the Psychotomimetic States Inventory was used to assess current schizotypal symptoms. It has been shown previously to be sensitive to acute cannabis-induced psychotomimetic effects. Participants rate statements describing their current experience from 0 (not at all) to 3 (strongly).

SPQ^17^—Trait schizotypy was assessed using the self-report SPQ. The SPQ is a series of 74 yes/no questions that assess nine characteristics of schizotypal personality disorder as defined by the *DSM-III-R*.^15^ Each item the participant responds to affirmatively is scored one point. Thus, participants were given schizotypy scores between 0 and 74, where higher scores indicate higher levels of schizotypy.

#### Spatial *N*-back task

This task taps spatial working memory with an increasing load. The participant was presented with a symbol (smiley face) in one of six spatial locations. A fixation cross remained in the centre of the screen throughout the task. When the next face appeared, they were required to indicate whether it was in the same location as the previous face in the 1-back version of the task, or the same location as the face two positions before (2-back). Each block consisted of 25 ‘match' and 25 ‘no-match' trials in random order, i.e. 50 trials in total, preceded by ten practice trials. All symbols were presented 5cm from the fixation cross. Each symbol was presented for 300ms with an inter-stimulus interval of 450 msec. The *N*-back data were analysed using signal detection theory. D prime (d′) scores were calculated as a sensitive measure of accuracy using the standardized difference between the hit rate (signal) and false alarms rate (signal+noise) using the equation: d′=*z* (H)−*z* (F).

#### Genotyping

DNA was obtained from cheek swabs of all participants who completed the assessments described above (422 cannabis users). DNA extraction was performed using standard phenol–chloroform methods. As the purpose of the study was to explicitly test for interaction at two specific single-nucleotide polymorphisms, a specific site within AKT1, and COMT, genotyping focused exclusively on the Valine158Methionine (Val158Met: (rs4680)) single-nucleotide polymorphism, reported in Caspi *et al.*^3^ and AKTI rs2494732 polymorphism reported by van Winkel *et al.*^8, 10^ Off the shelf Taqman assays for these polymorphisms are available as a kit (Applied Biosystems, Life Technologies, Paisley, UK). Genotype calls were discriminated on the basis of algorithmic membership of three clusters representing homozygote T/T, heterozygote C/T, and homozygote C/C genotype classes for AKT1, and A/A, A/G and G/G for COMT.

### Data analysis

Data were analysed with SPSS Statistics Version 21 (IBM, Armonk, NY, USA). Separate multiple hierarchical regressions were used to examine the impact of genotype, cannabis dependence (presence or absence, indicated by a Severity of Dependence Scale Score of 3 or greater) and the interaction (using centred variables to attempt to address the problem of multicollinearity) on acute psychotomimetic symptoms induced by cannabis, non-intoxicated psychotic symptoms and working memory after adjusting for confounding variables of gender and ethnicity. THC/cannabidiol ratio was also included. Genotypes at rs2494732 were coded to reflect an additive model.^18^ All the predictor values had tolerance and VIF values close to 1 excluding multicollinearity, and the assumption of independence of errors was tested with the Durbin–Watson statistic. For the analysis of moderation effects, variables were centred and then interaction terms added to the model to circumvent the problem of multicollinearity. To control for effects driven by ethnic minority populations, the significant analyses were repeated with only the ethnic majority (Caucasian).

## Results

### Sample

AKT1 rs2494732 gentoypes were in Hardy–Weinberg equilibrium (T/T=111; C/T=197; C/C=91; *χ*^2^ (2)=2.06; *P*=0.356). There were no differences in rs2494732 allelic distribution by gender (*χ*^2^ (2)=1.52, *P*=0.465) or cannabis dependence (*χ*^2^ (2)=3.281, *P*=0.194). COMT Val^158^Met genotypes were also in Hardy–Weinberg equilibrium (Val/Val=109; Val/Met=190; Met/Met=95; *χ*^2^ (2)=1.45, *P*=0.484) and again there were no differences in distribution by gender (*χ*^2^ (2)=5.54, *P*=0.136) or cannabis dependence (*χ*^2^ (2)=3.14, *P*=0.792).

There was no evidence of a correlation between AKT1 or COMT gentoype and years of cannabis use (*r*=0.007, *P*=0.895; *r*=−0.019, *P*=0.716) or days per month of cannabis use (*r*=0.014, *P*=0.784; *r*=0.006, *P*=0.902).

Data on demographics of the sample split by cannabis dependence are given in Table 1.

#### Acute psychotic symptoms induced by cannabis

**Figure 1:**
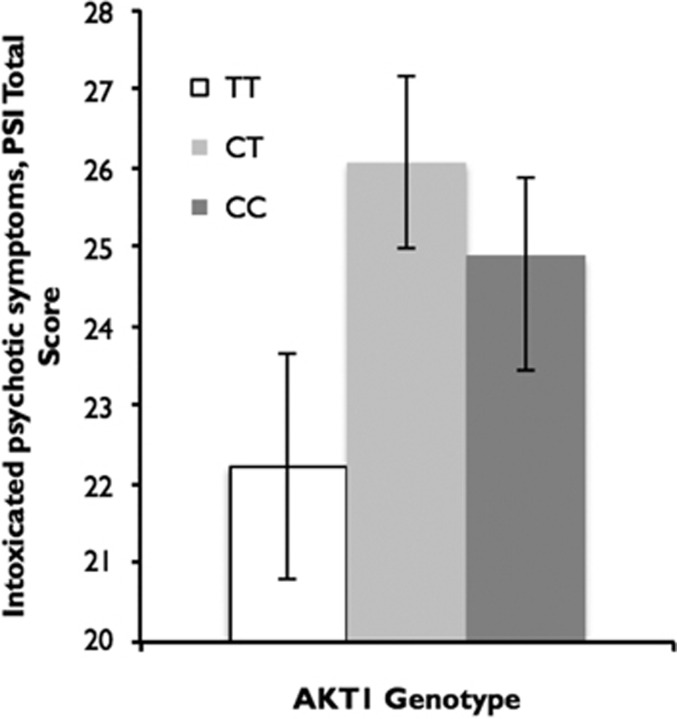
Acute psychotic symptoms, in total score when intoxicated with cannabis by AKT1 genotype, bar represent standard error.

A multiple hierarchical regression (See Table 2A and Table 2B; *R*^2^=0.198 , F(6,372)=2.07, *P*<0.001) found that the AKT1 genotype was a significant predictor of acute psychotomimetic symptoms associated with cannabis (*P*=0.015), with increasing ‘dosage' of C allele being associated with increased acute psychotomimetic symptoms induced by cannabis (see Figure 1). The other predictor was baseline schizotypal score (*P*<0.001). Years of cannabis use, gender, ethnicity (white versus other), cannabis dependence and COMT genotype were not significant predictors of acute psychotomimetic symptoms.

There was no moderating effect by years of cannabis use on the effect of AKT1 genotype on acute psychotic symptoms induced by cannabis, and the change in *R*^2^ from adding this term to the model was not significant (*R*^2^ change=0.001, F change (7,364)=0.067, *P*=0.795).

#### Sensitivity Analysis of AKT1 data

Including THC/cannabidiol ratio: A further multiple hierarchical regression (*R*^2^=0.17, F(7,184)=12.40, *P*<0.001) found AKT1 genotype to be a significant predictor of acute psychotic symptoms associated with cannabis (*P*=0.022), with increasing ‘dosage' of C allele to be associated with increasing acute psychotic symptoms induced by cannabis. The other predictor was baseline schizotypal score (SPQ; *P*<0.001). Years of cannabis use, gender, ethnicity (white versus other), cannabis dependence, THC/cannabidiol ratio and COMT genotype were not significant predictors of acute psychotic symptoms. THC and cannabidiol data are presented in Table 1.

There was no moderating effect by years of cannabis use on the effect of AKT1 genotype on acute psychotic symptoms induced by cannabis, and the change in *R*^2^ from adding this term to the model was not significant (*R*^2^ change=0.001, F change (2,173)=0.17, *P*=0.88).

Restricting analyses by dependence: Dependent users only: a further multiple hierarchical regression (*R*^2^=0.223, F(6,147)=7.01, *P*<0.001) found that AKT1 genotype was a significant predictor of acute psychotimimetic symptoms (*β*=0.162, *P*=0.035) along with SPQ score (*β*=0.416, *P*<0.001) and years of cannabis use (*β*=0.167, *P*=0.031).

Non-dependent users only: restricting the analysis to non-dependent users (*R*^2^=0.181, F(6,147)=7.00, *P*<0.001) found only SPQ to be predictive of acute psychotimimetic symptoms (*β*=0.398, *P*<0.001).

Analysis with only Caucasian participants: To control for effects driven by non-white ethnic minority groups, analyses were repeated including only the majority population, that is, Caucasian. The regression model was significant (*R*^2^=0.33, *P*<0.001). The pattern of results was identical to the mixed ethnicity sample: both SPQ total (*β*=0.482, *P*<0.001) and AKTI genotype (*β*=−0.192, *P*=0.02) were significant predictors of acute intoxicated psychotic symptoms.

#### Schizotypal symptoms when not intoxicated

The regression model with predictors of COMT and AKT1 genotype, cannabis dependence, years of cannabis use, adjusting for gender and ethnicity was of marginal significance (*R*^2^=0.03, F(7,364)=1.87, *P*=0.085). The only significant predictor of schizotypal symptoms when not intoxicated was cannabis dependence (*β*=−0.13, *P*=0.015).

#### Working memory performance

Intoxicated with cannabis: The model for performance on the 1-back part of the task (discriminability) was not significant (*R*^2^=0.017, F(7,361)=0.87, *P*=0.529; Table 3A and Table 3B). However for the 2-back part of the task, the model was significant (*R*^2^=0.042, F(7,365)=2.21, *P*=0.034) but with only gender emerging as a significant predictor (*β*=−0.14, *P*=0.010).

Non-intoxicated: The model examining accuracy, using the index of discriminability (d prime) on the 1-back part of the *N*-back task was marginally significant (*R*^2^=0.038, F(7,365)=1.79, *P*=0.088). The only significant predictors were ethnicity (*β*=−0.124, *P*=0.02) and a marginally significant predictor of COMT genotype (*β*=−0.08, *P*=0.091).

Discriminability on the 2-back part of the *N*-back task (d prime) was examined in a multiple hierarchical regression. The model was significant (*R*^2^=0.043, F(7,364)=2.30, *P*=0.027) but with only schizotypy (SPQ score) emerging as a significant predictor of working memory performance (*β*=−0.13, *P*=0.017).

## Discussion

To our knowledge, this study provides the first evidence that the acute psychotic effects of cannabis are predicted by variation at the rs2494732 locus of the AKT1 genotype. No evidence was found for an interaction of the COMT Val^158^Met genotypes with cannabis use, in producing psychotomimetic symptoms in this group of healthy cannabis users. Cannabis dependence predicted non-intoxicated schizotypal symptoms, but neither genotype had any impact on these. COMT Val^158^Met genotype had a marginal impact on performance on a working memory task when non-intoxicated and when memory load was low; however, at higher load, schizotypy was the only emerging predictor of performance. When intoxicated with cannabis, gender was the only predictor of working memory performance, with poorer performance in females at a high working memory load.

In the current study, which is the largest ever to be conducted on the acute response to cannabis, psychotomimetic symptoms while intoxicated were found to be predicted by variation at the rs2494732 locus of the AKT1 genotype in healthy young cannabis smokers, increasing with C allele dosage. These data are very important as acute psychotic response to cannabis is thought to be a marker of the risk of developing psychosis from smoking the drug.^1^ Two previous studies have implicated this polymorphism in the interaction with cannabis and psychosis,^9, 18^ but this work concentrated on individuals who were at familial risk of schizophrenia. This study is the first to demonstrate that the acute response to cannabis is modulated by AKT1 in otherwise healthy cannabis smokers. The mechanism for this modulation of acute effects may be through the interaction of AKT1 with dopamine.^2, 9^ Our sensitivity analyses suggested that these effects may be confined to dependent cannabis smokers but further investigation of these data with larger samples is required.

AKT1 codes for a protein that is a serine/threonine kinase, which has a variety of functions, one of which is as a signalling molecule downstream of the dopamine D2 (DRD2) receptor. Decreased AKT1 functionality may result in enhanced responses to DRD2 receptor stimulation.^19^ THC has been found to acutely induce dopamine release in rats^20, 21^ and in humans,^22, 23^ although not in all studies.^24^ Dopamine release is thought to occur via the blockade of cannabinoid 1 (CB1) receptors on GABAergic neurons that target pyramidal cells. These neurons normally exert an inhibitory effect on the firing of dopamine neurons that project back to the nucleus accumbens, so agonism of CB1 receptors by THC may produce increased dopamine release. This THC-mediated increase in dopamine release may be further exacerbated by decreased AKT1 functionality. Elevated levels of mesolimbic dopamine are known to have a role in the development of psychotic symptoms, potentially through disrupted salience attribution.^25^

In contrast to the role of variation at the rs2494732 locus of AKT1, this study found no support for the direct involvement of the functional polymorphism of the COMT gene in mediating acute psychotic response to cannabis. This is in contrast to one previous small-scale acute laboratory study giving acute THC to patients with schizophrenia,^26^ and other work that suggested that COMT may mediate the psychotomimetic risk of cannabis^3^ but in agreement with subsequent larger studies that failed to replicate these findings.^4, 27^ There was a marginal effect of COMT on working memory performance at a low load when not intoxicated. This polymorphism of COMT initially caused some excitement as several studies emerged demonstrating its association with working memory,^28, 29^ but this finding was not confirmed by meta-analyses,^30^ which suggested that this may be a case of publication bias.

Greater schizotypal symptoms predicted in poorer working memory performance on the more difficult section of the task among drug-free cannabis users. This echoes recent findings of poorer working memory in individuals high in schizotypy^31^ and indeed of the relationship between working memory performance and transition to psychosis.^32^ Working memory impairment is considered a central cognitive impairment in schizophrenia, and there is some evidence that such impairments are related to symptoms, particularly to negative symptoms.^33, 34^

Only gender predicted acute working memory impairment from cannabis, with greater impairment in females. Very few studies have examined gender differences in neurocognitive acute response to THC, with those that have using very small samples and in finding little evidence of gender differences.^35^ However, this study examined the acute effects of cannabis in over 400 cannabis smokers. There is an emerging preclinical literature that might explain this effect. CB1 density has been found to vary by gender, with animal studies reporting greater CB1 receptor density among males across several brain regions.^36, 37^ However, across their lifetime, adult female brains show increases in CB1 receptor density, with levels eventually surpassing those observed in males.^38^ Furthermore, greater CB1 de-sensitization after exposure to THC in the prefrontal cortex, hippocampus, striatum, amygdala and midbrain is seen in female adolescent rats.^36, 37^ Preclinical studies have also demonstrated that female rats preferentially metabolize THC to its most highly active metabolite, while male rats metabolize THC to multiple compounds.^39^ In combination, these findings may in part explain the finding of greater acute working memory impairment from cannabis in females. This also may partly be driven by gender differences in frequency of cannabis use. Users who smoked cannabis less frequently experienced stronger effects, and as there was a higher proportion of low frequency female cannabis users compared to males this may have contributed to the observed gender differences in working memory following the drug.

Strengths of this study include the large sample size for assessing acute cannabis effects. We also used independent verification of the cannabinoid content of the cannabis consumed and drug history. Further, the hypothesis-driven approach we took to genetic analysis was a strength, examining only loci implicated in previous studies and, therefore, circumventing some of the problems of type I error that have dogged earlier research. However, inevitably there are several limitations of the study. For the cannabis use data, while verifying past 3 months use with hair analysis, we inevitably relied on retrospective self-reports of drug use, which are particularly complicated as cannabis is known to acutely impair episodic memory. However, we opted to use years of cannabis use in this model as this was considered the most reliable to estimate. As we purposely recruited a young group of cannabis users, there was restricted variation in years used and future studies may investigate this further. We used a predominantly white Caucasian sample. However, it is unlikely that ethnic differences in allele frequency at rs2494732 biased the outcome of the study, as there was no difference between the frequency of rs2494732 alleles across the dichotomized ethnic groups. In addition, analyses with only Caucasian participants gave the same results to the analysis containing all ethnicities.

In summary, we found that the AKT1 rs2494732 C allele was associated with increased psychotomimetic symptoms after smoking cannabis. The other factor impacting on acute psychotomimetic response to cannabis was baseline schizotypy. Gender was the only factor to predict acute working memory impairment, with poorer performance in females. When drug free, cannabis dependence weakly predicted schizotypal symptoms and COMT genotype had a marginal impact on working memory, along with ethnicity. The findings of this study contribute to a recent and growing body of evidence suggesting that variation at the AKT1 locus confers details of the risk of cannabis smoking for schizophrenia. This is likely to be in the context of numerous other genetic variants, so the clinical utility at the moment is unclear. It is nonetheless encouraging that there is concordance between genetic influences on acute effects of cannabis and those mediating risk of psychosis. However, the fact that AKT1 is relevant to the biology of psychotic symptoms suggests that this might be a promising direction for novel therapeutics for cannabis-induced psychosis.

## Figures and Tables

**Table 1:** Demographic data by cannabis dependence group

	*Dependent cannabis users* *(*n=*169)*	*Non-dependent cannabis users* *(*n=*269)*
Age	20.77±1.82	20.43±177
WTAR score	41.07±7.93	44.74±4.77
		
*Gender*		
Female	43	88
Male	126	181
		
*Self-reported ethnicity*		
White Caucasian	110	189
Black Caribbean or African	5	7
Asian	33	45
Chinese/Japanese	3	11
Other/mixed	18	17
		
*AKT1 rs2494732 allelic frequency*		
T/T	43	52
C/T	74	143
C/C	52	74
		
*COMT Val*^*158*^*Met allelic frequency*		
Met/Met	68	40
Val/Met	133	78
Val/Val	68	51
Mean % THC level in cannabis smoked	9.56±4.53	9.69±4.75
Mean % CBD level in cannabis smoked	0.77±1.65	0.88±1.63
SPQ score	18.68±11.2	14.67±10.02
Years of cannabis use	5.01±2.45	4.73±2.38
Days per month cannabis is smoked	22.76±9.26	11.75±9.87
How long to smoke 3.5 g, days	2.35±2.34	9.9±16.58

Abbreviations: CBD, cannabidiol; COMT, catechol-O-methyltransferase; SPQ, Schizotypal Personality Questionnaire; THC, delta-9-tetrahydrocannbinol; WTAR, Wechsler Test of Adult Reading.

**Table 2A:** Hierarchical regression examining predictors of acute psychotomimetic symptoms induced by cannabis (*n*=422)

*Predictor*	B	*Standard error*	β	t
AKT1_rs2494732 Allele dosage	−2.682	1.098	0.119	2.443*
Gender	−1.808	1.736	−0.051	1.042
SPQ total	0.629	0.075	0.411	8.410**
Cannabis dependence	1.051	1.527	0.034	0.688
Ethnicity^a^	2.777	1.721	0.080	1.614
Years of cannabis use	−0.691	0.342	−0.100	2.019*
COMT_rs4680	−0.706	1.022	−0.033	0.691

Abbreviations: COMT, catechol-O-methyltransferase; SPQ, Schizotypyal Personality Questionnaire.

a Ethnicity defined as white Caucasian versus other.

*P<0.05; **P<0.01.

**Table 2B:** Hierarchical regression examining predictors of acute psychotomimetic symptoms induced by cannabis (including valid THC samples *n*=303)

*Predictor*	B	*Standard error*	**β**	t
AKT1_rs2494732	3.56	1.55	0.158*	2.29
Gender	−1.57	2.51	−0.043	0.627
SPQ total	0.564	0.104	0.372**	5.43
Cannabis dependence	−0.070	2.24	−0.002	0.031
Ethnicity	2.11	2.56	0.060	0.825
Years of cannabis use	−0.829	0.519	−0.114	1.60
COMT_rs4680	0.741	1.49	0.034	0.497
THC/CBD ratio	3.36	4.20	0.056	0.799

Abbreviations: CBD, cannabidiol; COMT, catechol-O-methyltransferase; SPQ, Schizotypyal Personality Questionnaire; THC, delta-9-tetrahydrocannbinol.

* *P*<0.05; **P<0.01.

**Table 3A:** Hierarchical regression predicting working memory (high load) when intoxicated

*Predictor*	B	*Standard error*	**β**	t
AKT1_rs2494732	0.095	0.084	−0.077	1.49
Gender	−0.355	0.133	−0.138*	2.66
SPQ total	−0.008	0.006	−0.077	1.48
Cannabis dependence	−0.176	0.117	−0.078	1.50
Ethnicity	−0.175	0.117	−0.078	1.50
Years of cannabis use	0.02	0.026	0.04	0.75
COMT_rs4680	−0.092	0.078	−0.060	1.17

Abbreviations: COMT, catechol-O-methyltransferase; SPQ, Schizotypyal Personality Questionnaire.

*P<0.05; **P<0.01.

**Table 3B:** Hierarchical regression predicting working memory (high load) when not intoxicated

*Predictor*	B	*Standard error*	**β**	t
AKT1_rs2494732	0.241	0.159	0.080	1.5
Gender	−0.222	0.136	−0.085	1.63
SPQ total	−0.014	0.006	−0.126*	2.39
Cannabis dependence	0.057	0.119	0.025	0.482
Ethnicity	−0.185	0.134	−0.074	1.38
Years of cannabis use	−0.027	0.026	−0.055	1.04
COMT_rs4680	−0.019	0.085	−0.012	0.222

Abbreviations: COMT, catechol-O-methyltransferase; SPQ, Schizotypyal Personality Questionnaire.

*P<0.05.
